# Microstructures and Corrosion/Localised Corrosion of Stainless Steels, Incoloy and Their Weldments in Nitrite-Containing Chloride Environments

**DOI:** 10.3390/ma17061336

**Published:** 2024-03-14

**Authors:** R. K. Singh Raman, W. H. Siew

**Affiliations:** 1 Department of Chemical and Biological Engineering, Monash University, Clayton, VIC 3800, Australia; whs6211@gmail.com; 2 Department of Mechanical and Aerospace Engineering, Monash University, Melbourne, VIC 3800, Australia

**Keywords:** weldments, duplex stainless steels, Incoloy, corrosion, localised corrosion

## Abstract

Prompted by the unexpected observation of the pitting of the weldments of a highly corrosion- and pitting-resistant duplex stainless steel, SAF2507, in chloride solutions with nitride addition, the pitting and corrosion resistance of SAF2507 and its weldments were investigated in chloride solutions with and without different levels of nitrite. The Incoloy 825 and 316L austenitic stainless steels were included for the purpose of developing a comparative appreciation. The microstructures of the weldments were characterised, and 316L showed a profound influence of nitrite addition in inhibiting pitting, while ‘meta-stable’ pitting transients that were clearly visible in the chloride solution without nitrite were absent when nitrite was added. Both the parent metal and the weldment of SAF2507 had similar pitting potential (E_p_) in 0.1 M NaCl without nitrite, which was the highest E_p_ among the three alloys tested. Additions of nitrite at low concentrations had an inhibitive effect on pitting, whereas higher nitrite contents had a deleterious effect on pitting resistance. On the other hand, Incoloy 825 showed a trend of E_p_ ennoblement with an increasing nitrite content of 0.1 M NaCl, and the weldment underwent greater ennoblement. Moreover, 316L showed a trend similar to Incoloy 825; however, the E_p_ ennoblements were significantly more pronounced for both the weldment and the base metal of 316L.

## 1. Introduction

Stainless steels (SSs) and nickel alloys (such as Incoloy) are commonly employed in applications for which corrosion/localised corrosion poses a concern. However, resistance to corrosion/localised corrosion can vary remarkably across the different alloy types. For example, duplex stainless steels (DSSs) and their advanced variants (super DSSs) are employed in applications that require high resistance to corrosion and localised corrosion (such as pitting). In addition to the optimised Cr and Mo contents in DSSs, their ferrite:austenite volume ratio of 1:1 contributes to their greatly superior resistance to pitting and stress corrosion cracking (SCC) in environments where traditional stainless steels (SSs) suffer from chloride pitting/SCC [1,2,3].

### 1.1. Electrochemical Aspects of Pitting

Pitting is a localised electrochemical dissolution as a result of a local disruption in the passive layer of a corrosion-resistant alloy, such as stainless steel. Pitting characteristics have a profound influence on the corrosion-assisted fracture of metallic materials, such as via stress corrosion racking (SCC), as will be obvious from one of the most accepted SCC mechanisms. The ‘dissolution-repassivation’ mechanism [4,5,6] necessitates the repetition of the following steps: (a) the applied stress causes the disruption of the passive film at the tip of a surface defect (such as machining notches/burrs), (b) the crack tip thus developed suffers from accelerated dissolution (since the tip acts as a small anode) and (c) the crack dip repassivates. Hence, the physical and chemical characteristics of the passive film that an alloy develops in a given corrosive environment are crucial for each step (a–c) and the sustenance of pitting/SCC [7]. The chemical/electrochemical nature of the corrosive environment dictates the physico-chemical characteristics of the passive film (and pitting/SCC susceptibility), and even a minor variation in the environment can have a profound impact. For example, the addition of just 0.4 M of sulphide to a 3.5 M alkaline solution enhanced the SCC rate of steel by 25 times [8,9]. However, the profound roles of such minor variations are often ignored in addressing corrosion-assisted material degradation/failures. Hence, it is not uncommon to come across corrosion-assisted failures in industry in spite of industry representatives claiming to have implemented laboratory data (that were actually generated using unrepresentative and simpler test environments).

### 1.2. Pitting of Corrosion-Resistant Alloys and Their Weldments

Duplex stainless steels (DSSs) possess an austenite:ferrite volume ratio of 1:1, which enables their excellent resistance to pitting/SCC in high-chloride environments where common austenitic SSs would fail [1,2,3]. SAF 2507 is a highly alloyed duplex stainless steel with exceptionally high resistance to pitting (pitting resistance equivalent number (PREN) >40). The temperature limits for pitting resistance in a chloride environment can be as high as 150 °C for DSS [10], whereas such limits for the common SS grades can be much lower (around 50 °C) [11,12], suggesting DSSs to be pitting-resistant up to much higher temperatures. The superior resistance of DSSs to chloride pitting/SCC is attributed to the electrochemical and mechanical influences of the constituent austenite and ferrite phases. Austenite is susceptible to pitting, but it is cathodically protected via ferrite [1,2], hence the superior pitting resistance of DSSs. When subjected to tensile loading, ferrite undergoes compressive stress (due to the ‘Keying Effect’ [13] in the dual-phase structure), hence the superior resistance of DSSs to SCC (since SCC occurs only under tensile loading). The superior pitting/SCC resistance of DSSs to the most commonly used stainless steel type (austenitic stainless steels (ASSs)) is also attributed [14] to their resistance to sensitisation (i.e., the formation of chromium carbide at the alloy grain boundaries, leaving the neighbouring area lean in Cr and susceptible to intergranular corrosion (IGC) and intergranular SCC (IGSCC)). ASSs form different secondary precipitates (*viz*., σ, χ, α′ and Cr_2_N); however, σ is the most detrimental to resistance to pitting/SCC [15]. Sensitisation (and the resulting IGC/IGSCC) is a particular concern for weldments of ASS (with sufficient carbon contents) because the steel is subjected for extended durations to such thermal conditions that cause sensitisation. On the other hand, the microstructure of DSSs is less favourable for the required diffusion characteristics for sensitisation/IGC/IGSCC. The following description elaborates upon the electrochemical aspects of the pitting and SCC of corrosion-resistant alloys and the role of microstructural variations in welded alloys.

The addition of chromates, sulphates, molybdates and nitrates to a chloride solution has been reported to inhibit the pitting of stainless steels [16,17,18,19,20,21,22,23,24]. On the other hand, there are also examples of the profound roles of minor environmental variations in enhancing the pitting susceptibility of highly corrosion-resistant stainless steels and their weldments, such as the pitting (and SCC) of the weldments of a stripping column that was constructed from a super duplex stainless steel (SAF2507). Pitting was found to have accelerated when a nitrite compound was used as an alternative chemical for arresting the polymerisation reactions in the stripping step (note, aqueous chloride and a moderately high temperature (~90 °C) constituted the chemical environment inside the column). Newman and Ajjawi [17] found a nitrate addition to an aqueous chloride solution to facilitate passivation; however, the enhanced passivating effect depended on the electrochemical potential and the chloride-to-nitrate ratio. They also suggested in their subsequent study that the electro-reduction of nitrate may generate nitrogen, thereby enhancing pitting resistance (note that this proposition is consistent with the enhanced pitting resistance of nitrogen-containing SSs). In contrast, the nitrite addition to the aqueous chloride environment was found to accentuate the pitting of the DSS weldments in the stripping column, as described earlier. The role of a nitrite addition in the SCC of austenitic SSs has also been investigated [25]. The super duplex SS SAF2507 is reported to be immune to pitting in an aqueous chloride environment up to 300 °C [11], and hence, it was surprising to observe its pitting in the stripping column that operated at just 95–110 °C. However, the instances of pitting were localised to the weldments in the column. For their direct industrial relevance, microstructural variations in DSSs and their influence on corrosion continue to be a topic of research interest [26]. The welding of SSs can incorporate such changes in microstructure and microchemistry that make the steel more susceptible to pitting (and SCC) in chloride environments [27]; for example, the welding of common austenitic SSs can cause sensitisation or the formation of σ phase, which are known to enhance pitting susceptibility. In this respect, it is critical to understand whether welding imparted any deleterious changes to the DSS microstructure (i.e., in addition to the detrimental role of a NO_2_^−^ addition). Here, it may be useful to reiterate that, unlike ASSs, DSSs are resistant to sensitisation or σ phase formation. However, improperly carried out welding can incorporate deleterious changes to the DSS microstructure that can jeopardise the basic characteristics responsible for the excellent pitting resistance of DSS; i.e., improper welding can disturb the optimum austenite:ferrite volume ratio of 1:1 [11,13] in the welds and the adjacent heat-affected zone (HAZ). Therefore, the welding of DSS necessitates highly skilled personnel who can suitably engineer the weld metal and HAZ microstructure and retain the optimum austenite:ferrite volume ratio of 1:1, such as by employing low-energy input processes (e.g., electron-beam or laser welding).

In light of the preceding descriptions on the roles of nitrite/nitride addition to the aqueous chloride environment and the role of microstructural variations in pitting, this study investigated the corrosion and pitting characteristics of the duplex stainless steel SAF2507. To develop a comparative appreciation, the study also included the investigation of a common austenitic stainless steel (316L SS) and another highly corrosion-resistant alloy, Incoloy 825.

## 2. Experimental Methodologies

### 2.1. Test Alloys

The corrosion-resistant alloys and their weldments investigated in this study are the SAF 2507 (UNS S32750) super duplex stainless steel and Incoloy 825 (UNS N08825); both have been employed in nitrite-containing aqueous chloride environments (such as the one in the stripping column, where pitting/SCC was found to occur, as described earlier). The austenitic stainless steel 316L (UNS 31603), an SS grade that is widely used in industrial applications, was included for comparison. The alloys were received in the form of plates (500 mm × 300 mm × 10 mm). The chemical compositions of the alloys, along with their respective typical compositions, are presented in Table 1.

SAF2507, a super duplex stainless steel (austenitic–ferritic), provides high strength and resistance to chloride stress corrosion cracking. It has higher chromium (~25%) and molybdenum (~4%) contents than duplex stainless steels. It also has low carbon contents (>0.3%), which avoids sensitisation during the thermal cycles that occur in the course of welding, hence the alloy’s resistance to intergranular corrosion. The high chromium and molybdenum contents make SAF2507 highly resistant to uniform corrosion, pitting and crevice corrosion. The equal volume ratio of austenitic and ferritic phases imparts a remarkable resistance to pitting/SCC. Ferrite cathodically protects the more susceptible austenite, whereas SCC crack propagation is impaired via austenite; being considerably softer (than ferrite), austenite accommodates the majority of the strain.

Incoloy 825 is a nickel–iron–chromium alloy with considerable additions of molybdenum and copper. It possesses remarkable corrosion resistance. The high chromium content confers remarkable corrosion resistance, and the molybdenum content provides high resistance to pitting, whereas the higher nickel content (compared to Alloy 800) accounts for the greater resistance to chloride SCC.

Meanwhile, 316L is a *low-carbon* variety of type-316 austenitic chromium–nickel stainless steel. Nickel acts as an austenite-stabilising agent and is balanced against the chromium content to minimise the formation of delta ferrite, which is known to decrease the pitting resistance of steel. The addition of molybdenum improves the overall corrosion resistance, as well as the pitting resistance in chloride environments. Low carbon content minimises carbide precipitation when exposed to elevated temperatures such as during welding, thus reducing the susceptibility to localised corrosion due to sensitisation.

### 2.2. Weldments of Test Alloys and Their Microstructures

**Figure 1:**
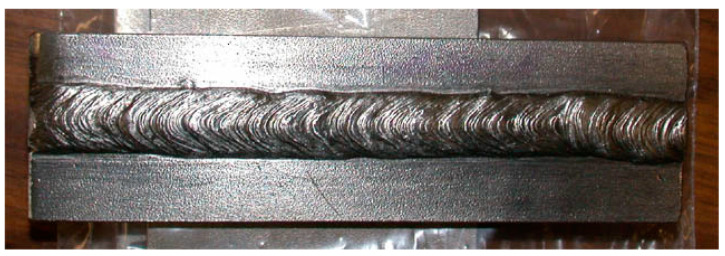
Weld plate ready for machining into rectangular weldment specimens.

Weldments of the three alloys were prepared via an arc welding process (a gas tungsten arc welding (GTAW) or manual metal arc welding (MMAW) process) by a speciality welding company (John Beever Pty. Ltd., Melbourne, VIC, Australia). A section of a typical welded plate is shown in Figure 1.

The chemical compositions of the welding filler wires are described in Table 2. High-silicon-content filler wires were used for the welding of DSS 2507 and Incoloy 825 to improve the washing and wetting behaviour of the weld metal. Silicon also has the ability to help with de-oxidation and in the formation of ferrite in a weld, which decreases hot-cracking sensitivity. The higher nickel and molybdenum contents (compared to their respective base metals), which are typical in the weld filler composition for such alloys, allow for microsegregation in the weld metal, heat-affected zone (HAZ) and fusion zone.

The microstructures of the weldments were characterised by optical microscopy. Microscopy also helped in establishing the quality of the weldments and their research-worthiness. Sections of the weldment and the base alloy were mounted in cold-form epoxy resin. Prior to mounting, a screw was installed on one side of the specimen for the purpose of establishing electrical conductivity, which is required for the electrochemical etching of the polished specimens. The mounted specimens were subjected to grinding and polishing up to a 1 μm surface finish using grinding papers of successive fineness (Grades 180, 400, 800, 1200 and 2400), followed by polishing with polishing cloths charged with diamond (3 and 1 μm) suspension fluid. All polished samples were rinsed with distilled water, ultrasonically cleaned in ethanol, dried and stored in a desiccator. Because the test alloys are highly corrosion-resistant (particularly, DSS and Incoloy 825), it required electrochemical etching to reveal the microstructural features of the weldments. In fact, it required optimisation of the voltage, current and electrolyte, as described in Table 3, in order to achieve distinct microstructural features. Duly etched specimens were rinsed in running water, cleaned in ethanol, dried and stored in a desiccator.

### 2.3. Pitting Tests

ASTM G48-92 allows a direct qualitative comparison of the susceptibility of metals and alloys to pitting corrosion when exposed to an acidic chloride solution. Immersion in 6% ferric chloride (FeCl_3_) at a temperature of 50 °C is considered a severe test for any ferrous alloy, as FeCl_3_ is a strong oxidiser, which, in combination with an acidic solution, high chloride concentrations and elevated temperatures, provides a suitable environment for pit nucleation and subsequent stable growth, and even highly corrosion-resistant alloys suffer pitting attack in a relatively short time. A visual examination with the aid of an optical microscope usually permits a clear assessment of the relative susceptibility of alloys to pitting.

A section of the weldment of each alloy (similar to the one shown in Figure 1) was cut using EDM (electro-discharge machining) into coupons (2.5 mm × 10 mm × 11 mm) and polished progressively to a 1200-grit finish on both sides. These sections of composite weldments contained all three regions of the weldments, viz. the base metal, HAZ and weld metal. A small hole drilled at one corner away from the weld enabled the use of a nylon string for suspending the coupon in the test environment, avoiding possible crevice effects which could have arisen due to contact with the glass walls of the container if the coupons had been simply placed in the corrosion test vessel. Each specimen was subjected to the same level of polishing to ensure a reproducible and consistent surface finish for accurate comparison. Each coupon was suspended in a test solution of 6 wt.% FeCl_3_ maintained at 50 °C for 72 h, after which each was ultrasonically cleaned in 10% nitric acid (to remove corrosion products), degreased with acetone and finally dried with compressed air. A qualitative assessment could be made visually (using the unaided eyes and optical microscopy) of the extent of the pitting attack on the alloy weldments.

### 2.4. Immersion Tests and Corrosion Rates

ASTM G31-72 is commonly used for quantitative screening based on the measurement of the weight loss of alloy specimens exposed to a given corrosive environment. It was first decided to employ 6% FeCl_3_ as the test solution to simulate a severely corrosive environment, followed by tests to evaluate the effects of nitrite additions on the corrosion rates of the test specimens. For these tests, coupons of both the parent (base) metal and the composite weldment were used.

Both faces of the coupons of the parent metal and the weldment were polished to a 1 μm finish. The polished coupons were cleaned and weighed carefully on an electronic balance with an accuracy of 1 mg. Securely suspended from one corner with nylon strings, the coupons were immersed in a corrosive test solution comprising 6% FeCl_3_ only (the control) and 6% FeCl_3_ with three different concentrations of nitrite anions achieved by dissolving specific amounts of NaNO_2_ salt crystals in order to produce solutions with chloride:nitrite ratios in the desired range (as described in Table 4). The temperature of the test solution was maintained at 50 °C, and the test duration was a conservative 72 h. At the end of the test, the samples were carefully removed and ultrasonically cleaned in an inhibited acid solution for 30 min, followed by 15 min drying in an oven at 90 °C. The specimens were weighed again on an electronic balance to determine the mass loss due to corrosion, and the corresponding corrosion rates were calculated.

Corrosion rates were calculated from the weight loss, based on the ASTM G31-72 method as follows:

$${\mathrm{Corrosion}\;\mathrm{rate}\;(\mu g/m}^{2}s)\;=\;\frac{K*W}{A*T}$$

where *K* = 2.78 × 10^6^ (constant), *W* = mass loss in g, *A* = area in cm^2^ and *T* = exposure time in hours.

### 2.5. Potentiodynamic Polarisation

Potentiodynamic polarisation (PDP) is commonly employed for the characterisation of pitting and corrosion. The potential of the test material (a working electrode) was dynamically swept using a computer-controlled potentiostat, resulting in a current response curve displaying both cathodic and anodic regions. The reference electrode was a saturated calomel electrode (SCE) with a stable potential of 0.244V (vs. a standard hydrogen electrode). The counter-electrode utilised was a high-purity graphite rod. PDP tests were conducted at a scan rate of 0.5 mV/s. The corrosion cell was filled with the test electrolyte and heated to the desired temperature, at which point the working electrode was lowered carefully into the test electrolyte. The working electrode surface was then held for 60 min for the stabilisation of open circuit potential (OCP).

## 3. Results and Discussion

### 3.1. Microstructures of Weldments

#### 3.1.1. Duplex Stainless Steel, SAF2507 (UNS S32750)

**Figure 2:**
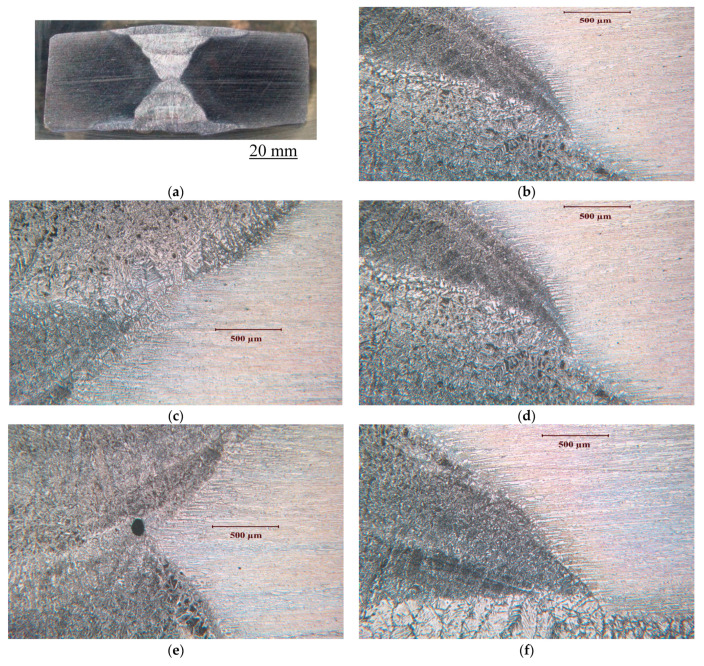

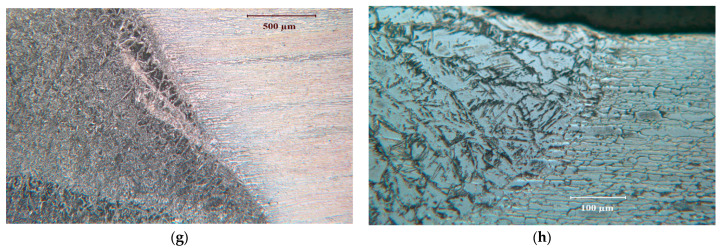
(**a**) Cross section and microstructural variations at different locations in the multi-pass weld of SAF 2507: (**b**–**h**) examples of a few locations.

Figure 2 illustrates the cross section and microstructural variation of the welded SAF 2507 sample.

**Figure 3:**
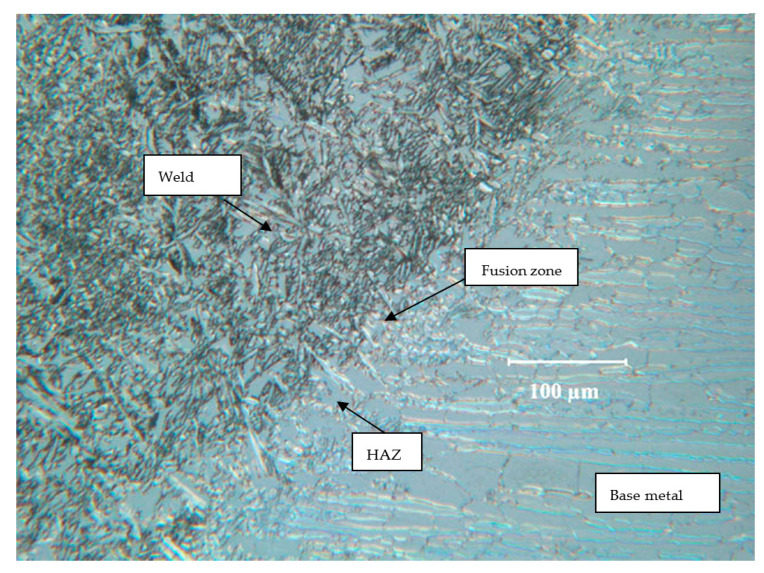
Typical microstructural features in the weldment of SAF2507.

Multi-pass welding creates variety of variations in the microstructure, as seen in different micrographs in Figure 2. Figure 3 presents the description of a typical microstructural variation in the SF2507 weldment. Elongated austenite and ferrite grains in the base metal originated from the prior extrusion. However, the fresh grains that formed upon crystallisation during the solidification of metal in the weld and recrystallisation in the heat-affected zone (HAZ) were largely equiaxed.

#### 3.1.2. Incoloy 825 (UNS N08825)

**Figure 4:**
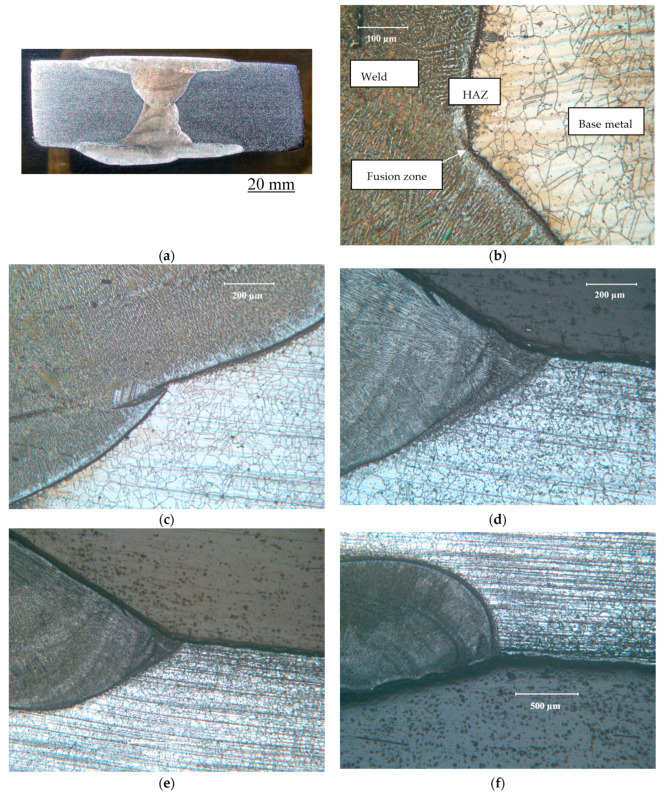
(**a**) Cross section and microstructural variations at different locations in the multi-pass weld of Incoloy 825: (**b**–**f**) examples of a few locations.

Figure 4 illustrates the cross section and microstructural variation in the welded Incoloy 825 sample. Multi-pass welding creates a variety of variations in the microstructure, as seen in different micrographs in Figure 4. Because of the high Ni content, the alloy has an austenitic structure; however, the variations in the grain morphology across the weldment, as observed in the micrographs, result from the difference in cooling characteristics in different regions.

#### 3.1.3. Stainless Steel 316L (UNS S31603)

**Figure 5:**
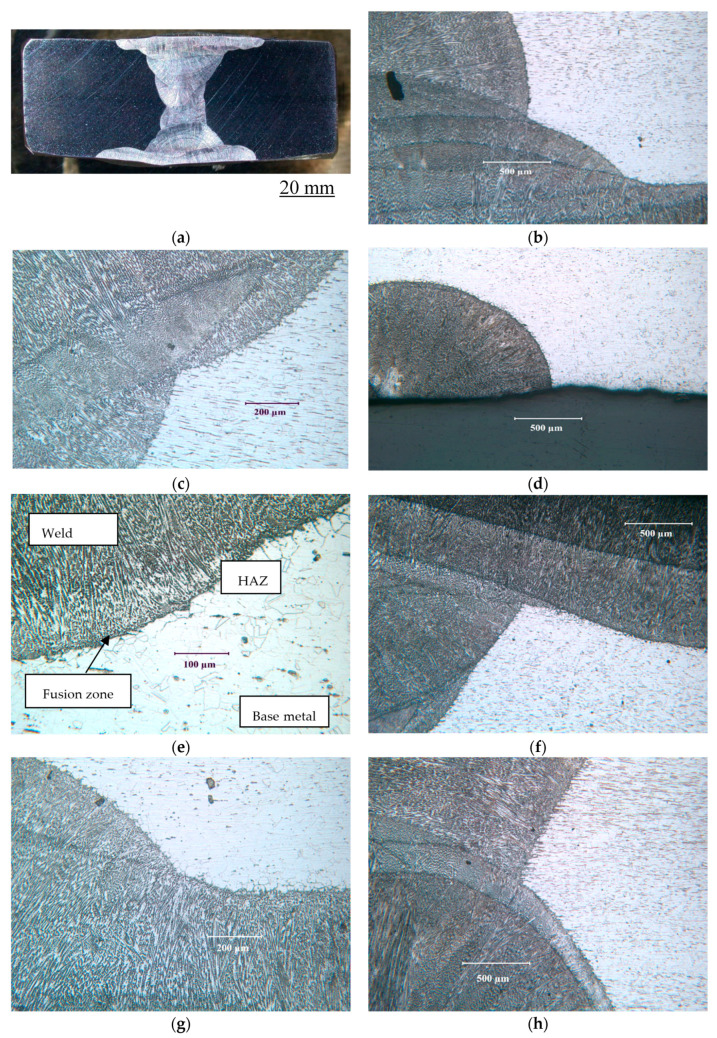
(**a**) Cross section and microstructural variations at different locations in the multi-pass weld of 316L: (**b**–**h**) examples of a few locations.

Figure 5 illustrates the cross section and microstructural variation in the welded 316L sample.

### 3.2. Pitting and General Corrosion Resistance of the Alloys and Their Weldments

In order to develop a broad ranking of the pitting resistance of the weldments of the three alloys, standard pitting tests were performed in the relatively accelerating conditions of the ferric chloride solution. Following the broad ranking, further tests focussed on the role of nitrite additions.

#### 3.2.1. Tests in Plain FeCl_3_

**Figure 6:**
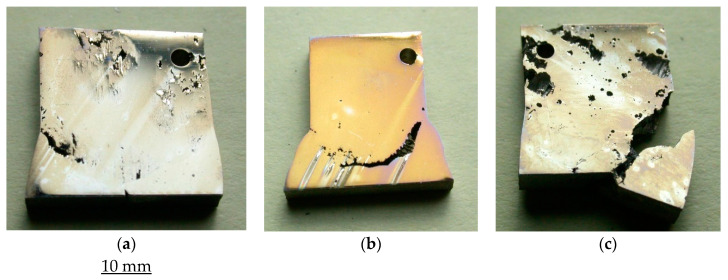
Weldments of alloys after immersion in 6% FeCl_3_ at 50 °C for 72 h: (**a**) SAF 2507, (**b**) Incoloy 825 and (**c**) 316L.

Tests of the alloy weldments suggest the superior resistance of the parent metal, HAZ and weld metal of Incoloy 825 to pitting and corrosion in 6% FeCl_3_ at 50 °C compared to the corresponding regions of the weldments of SAF 2507 and 316L, as shown in Figure 6. However, the HAZ of the weldments of all three alloys are the most susceptible to attack.

As shown in Figure 6, the weld metal and base metal of SAF 2507 and Incoloy 825 appear to be highly resistant to pitting attacks (compared to those of 316L); however, some areas of the base metal of SAF 2507 suffered some corrosion. On the other hand, the HAZ of SAF 2507 showed greatly superior resistance compared to that of Incoloy 825 HAZ. While both the weld metal and parent metal of 316L suffered from an extensive corrosion attack (Figure 6c), as expected for the significantly lower chromium and molybdenum contents of the steel, the attack was particularly severe in the HAZ, to the extent that the attack on the HAZ resulted in the separation of the weld metal and the parent metal.

#### 3.2.2. Role of Nitrite Addition in Corrosion and Pitting

The corrosion of the overall weldments and base metals of the three alloys was compared upon the immersion of their coupons in 6% FeCl_3_ at 50 °C. For an investigation of the role of nitrite in corrosion susceptibility, immersion tests were also conducted in FeCl_3_ solutions with three different concentrations of nitrite, as described in Table 4.

**Figure 7:**
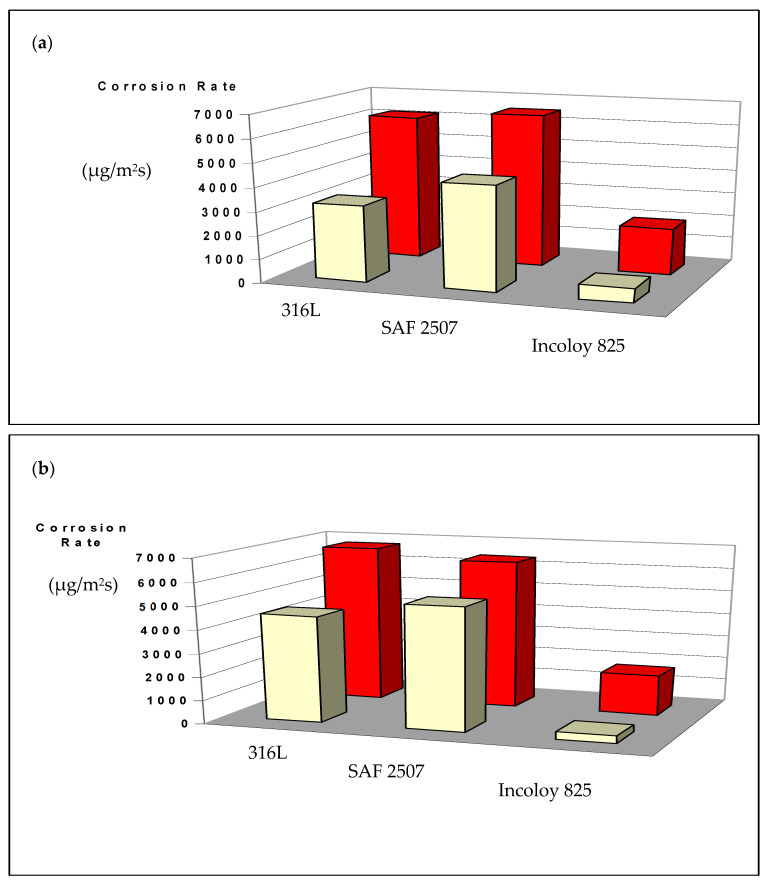

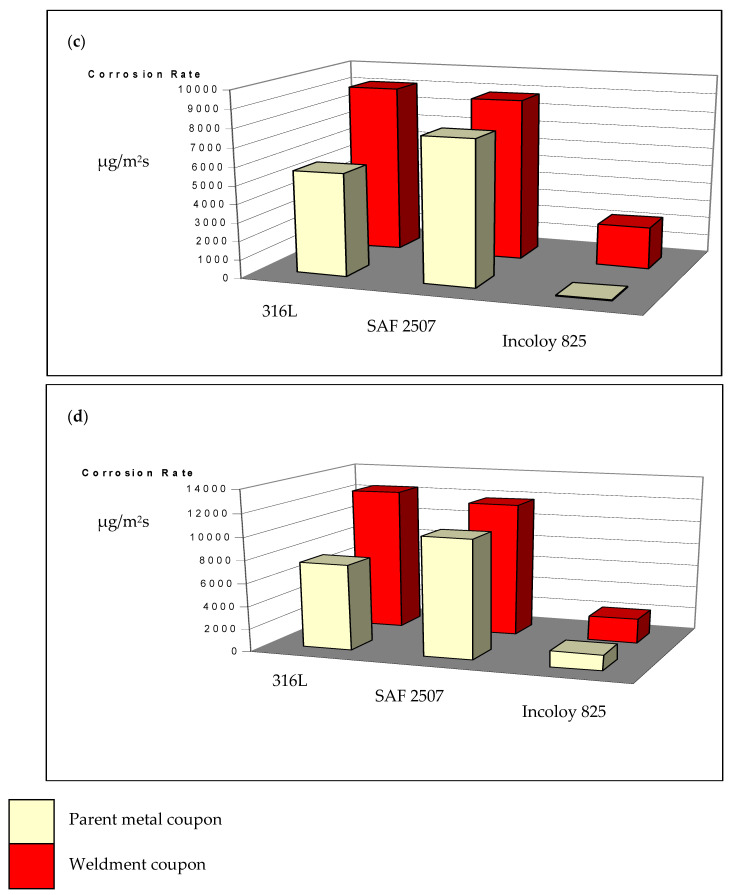
Weight loss of the parent metals and weldments of SAF 2507, Incoloy 825 and 316L in a 6% FeCl_3_ solution with different levels of nitride additions (described in Table 4): (**a**) 0 ppm, (**b**) 3963 ppm, (**c**) 5903 and (**d**) 7872 ppm.

As shown in Figure 7, the corrosion rate of the parent metal (3.5 × 10^3^ μg/m^2^ s) and weldment (6 × 10^3^ μg/m^2^ s) of SAF 2507 were, respectively, similar to those of the 316L stainless steel in FeCl_3_ solutions with and without a nitrite addition; however, SAF 2507 showed a greater resistance to pitting (as shown in Figure 6). The corrosion resistance of both the parent metal (5 × 10^2^ μg/m^2^ s) and the weldment (1.5 × 10^3^ μg/m^2^ s) of Incoloy 825 was much lower (than the corresponding samples of SAF 2507/316L). For each alloy, the corrosion rate of the parent metal was lower in each environment, which may possibly be attributed to the greater pitting susceptibility of the HAZ in the weldment of each alloy (Figure 6).

Scrutinising the data in Figure 7a–d suggests that, as the nitrite concentration was gradually increased from 0 ppm to 7872 ppm, the corrosion rates of SAF 2507 and 316L increased, whereas the corrosion rate of Incoloy 825 remained largely unchanged with increasing nitrite levels. This is consistent with the industry experience of the stripping column (described in the ‘Introduction’ section), when constructed from Incoloy 825, generally suffering little corrosion damage.

**Figure 8:**
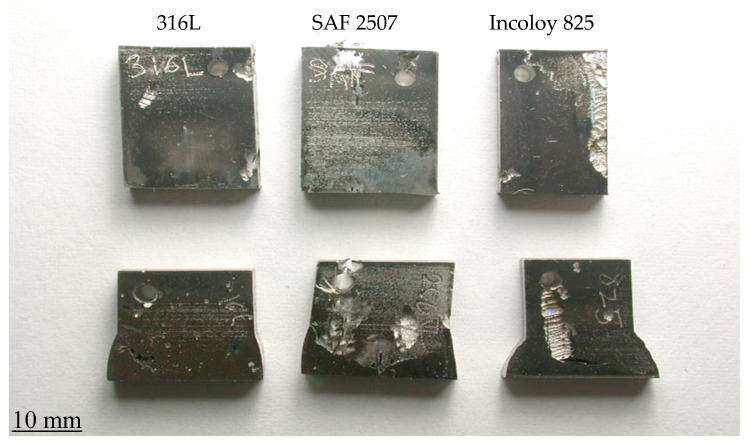
Appearance of coupons of parent metals and weldments of SAF 2507 and Incoloy 825 and 316L after exposure to a 6% FeCl_3_ solution (with no nitrite) at 50 °C for 72 h (**Top row**: parent metals; **bottom row**: weldments).

**Figure 9:**
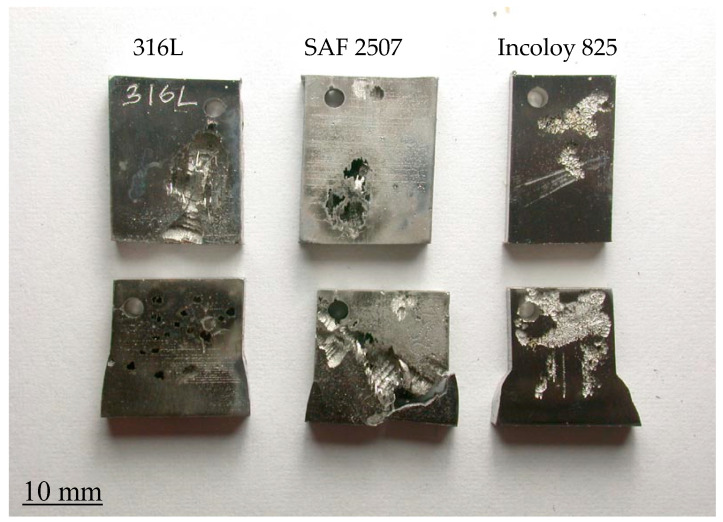
Appearance of coupons of parent metals and weldments of SAF 2507 and Incoloy 825 and 316L after exposure to a 6% FeCl_3_ solution (with 7872 ppm nitrite) at 50 °C for 72 h (**Top row**: parent metals; **bottom row**: weldments).

Figure 8 shows the visual appearance of the duly cleaned coupons of parent metals and weldments after immersion in plain FeCl_3_, whereas Figure 9 shows the coupons after immersion in FeCl_3_ with 7872 ppm nitrite. Corrosion attacks appeared to be particularly severe on the weldment coupons of SAF 2507. Meanwhile, the 316L coupons also suffered a considerable degree of undercut pitting (which is not clearly visible in the photograph). The addition of 7872 ppm nitrite to FeCl_3_ caused very severe corrosion damage to the SAF 2507 weldment coupon (Figure 9). Consistent with the corrosion rate data (Figure 7), corrosion damage to the Incoloy 825 coupons remained less severe even with increasing nitrite concentrations (and it was generally limited to shallow surface attacks).

SAF 2507 is generally considered to have very high resistance to pitting corrosion, especially in chloride environments, as is evident from its pitting resistance equivalent number (PREN) [12]. The PREN of an alloy is calculated from the chromium, molybdenum and nitrogen content based on the following equation:

PREN = % Cr + 3.3 (% Mo) + 16 (% N)

The PREN of the alloys in this investigation are presented in Table 5.

Table 5 clearly shows that SAF 2507 has a higher PREN compared to both Incoloy 825 and 316L. However, as shown in Figure 6, Figure 7, Figure 8 and Figure 9, the pitting and corrosion resistance of SAF 2507 were found to be inferior to what is widely believed for the alloy. The weight loss and pitting measurement conducted in 6% FeCl_3_ without nitrite indicated that the parent metal of SAF 2507 was slightly more susceptible than the parent of 316L and considerably more susceptible than the parent of Incoloy 825. This suggests that, although SAF 2507 displays excellent initial resistance to pitting corrosion, the alloy does not continue to have the same inherent resistance once stable pitting starts. Once stable pitting commences, the corrosion rate of SAF 2507 is comparable to that of alloy 316L. Similar trends were also observed for the weldment coupons of the three alloys. Incoloy 825 displayed greatly superior corrosion resistance to both 316L and SAF 2507. Considerably greater nickel content constitutes the major chemical difference in the composition of Incoloy 825 vis-à-vis that of 316L and SAF 2507. It will be interesting to know whether other high-Ni alloys, such as Sanicro, also show superior corrosion resistance.

#### 3.2.3. Potentiodynamic Polarisation to Characterise Pitting and Corrosion

Potentiodynamic polarisation (PDP) is commonly employed for the accelerated electrochemical characterisation of pitting and corrosion mechanistics. To characterise the critical pitting temperature (CPT), PDP tests were conducted using a 0.1 M NaCl electrolyte at different temperatures of up to 90 °C (which constitutes the chloride content and temperature similar to the environment in the stripping column). The CPT for each alloy was established by running polarisation scans at different temperatures to identify the temperature above which pitting could be electrochemically identified, such as the appearance (in polarisation curves) of a sudden ‘breakaway’ in corrosion current densities at a specific potential known as the pitting potential, E_pit_ or E_p._

**Figure 10:**
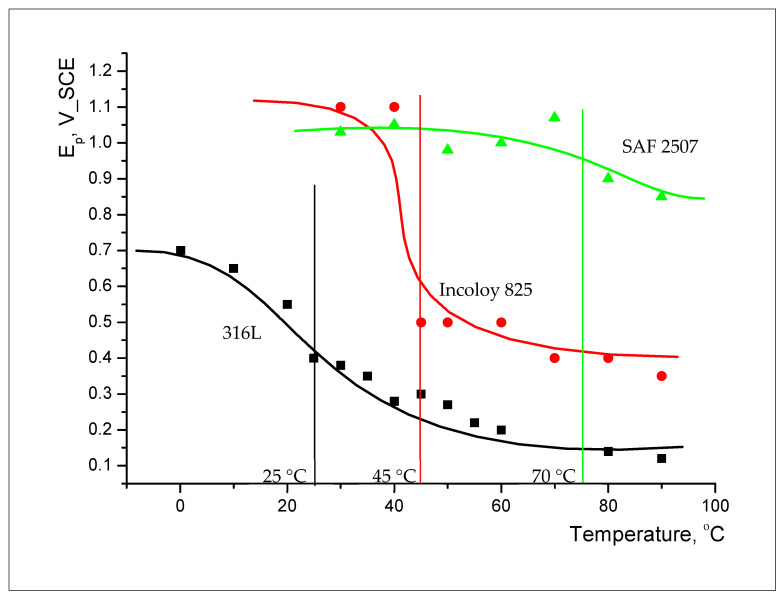
Pitting potential (E_p_) vs. temperature for SAF 2507, Incoloy 825 and 316Lin plain 0.1 M NaCl.

Figure 10 presents a plot of E_p_ vs. temperature for each alloy. A decrease in E_p_ with an increasing temperature identifies CPT. The following CPT of the alloys in 0.1 M NaCl were found: SAF 2507, 70 °C; Incoloy 825, 45 °C; and 316L, 25 °C.

**Figure 11:**
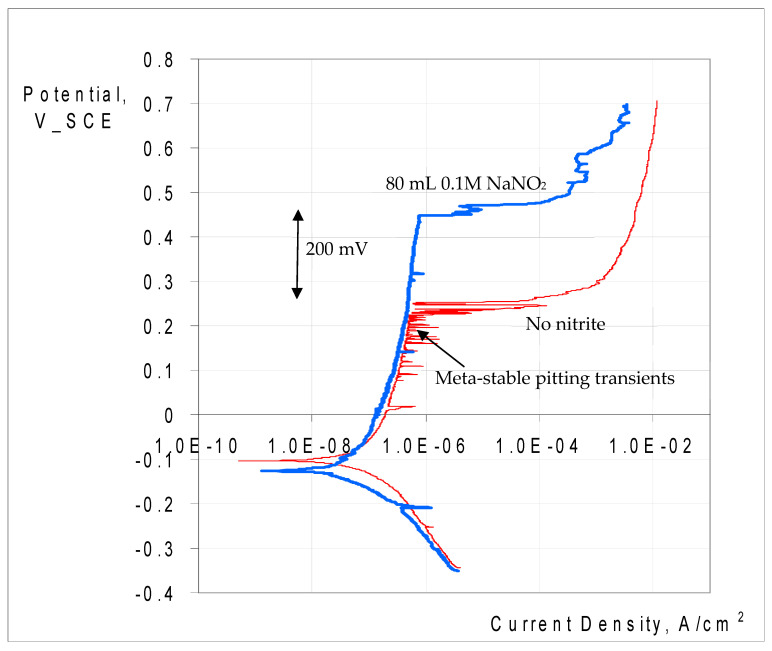
Potentiodynamic polarisation of 316L in 0.1 M NaCl at 60 °C with and without nitrite addition (80 mL 0.1 M NaNO_2_).

The role of nitrite additions to 0.1 M NaCl in CPT was characterised by carrying out PDP at temperatures higher than CPTs for the three alloys. The test temperature for SAF 2507 and Incoloy 825 was 90 °C (which was decided upon on the basis of the operating temperature in the stripping column constructed out of the two alloys and the occurrence of pitting in the column). However, 316L was tested at 60 °C to investigate and establish the role of a nitrite addition in the case of an alloy that was the most susceptible to pitting at elevated temperatures. Indeed, 316L showed a profound influence of nitrite in inhibiting pitting, as is evident in Figure 11, which shows an increase in E_p_ of 316L in 0.1 M NaCl by 200 mV when nitrite was added to the electrolyte. However, the nitrite addition caused very little change in the corrosion potentials, E_corr_ (i.e., around −100 mV vs. SCE). The corrosion current densities (i_corr_) derived from the Tafel extrapolations, too, were similar. Another striking feature is the absence of ‘meta-stable’ pitting transients when nitrite was present in the test solution, whereas the meta-stable pits are clearly visible in the polarisation scan in 0.1 M NaCl without nitrite.

PDP tests were also carried out for the parent metals and weldments of the three alloys in 0.1 M NaCl with additions of different nitrite concentrations at temperatures significantly above their respective pitting temperatures (90 °C for SAF 2507 and Incoloy 825 and 60 °C for 316L).

**Figure 12:**
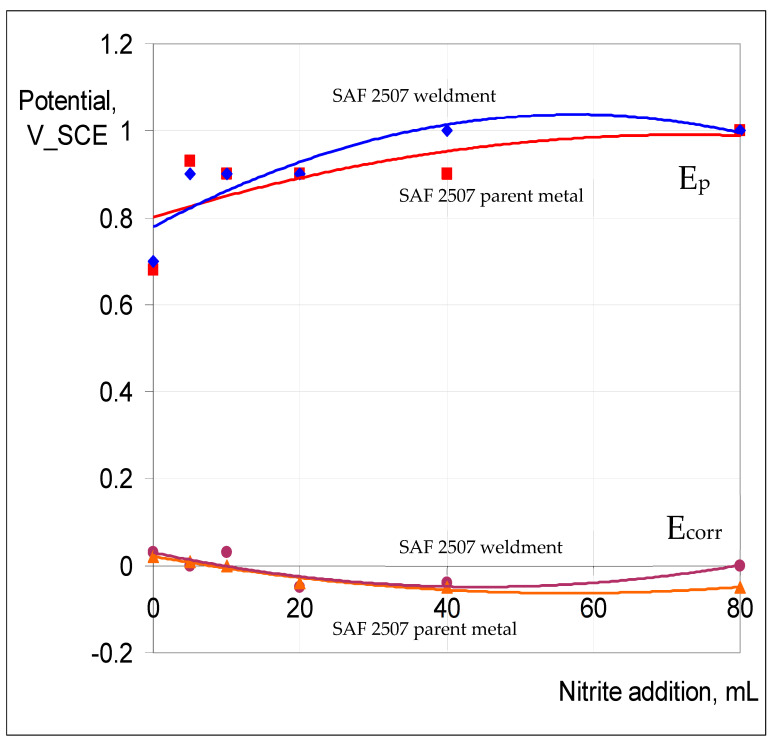
E_p_ and E_corr_ vs. nitrite concentration in 0.1 M NaCl for SAF 2507 parent metal and weldment at 90 °C.

Figure 12 presents the summaries of the variation in E_p_ and E_corr_ for the parent metal and weldment of SAF 2507 exposed to 0.1 M NaCl at 90 °C with increasing NaNO_2_ additions. Both the parent metal and weldment had similar E_p_ (~700 mV) in 0.1 M NaCl without nitrite, which was the highest E_p_ among the three alloys tested. The additions of 20 mL and 40 mL of 0.1 M NaNO_2_ appear to have had an inhibitive effect on pitting, whereas a further increase in the nitrite content (to 80 mL) seems to have compromised the pitting resistance. This behaviour is consistent with the reported literature [28] in which the passivation characteristics of the duplex stainless steel in a chloride solution were found to improve, and E_p_ was found to increase with the increasing nitrite content of a chloride solution from 0–100 ppm, whereas a further increase to 1000 pm began to significantly perturb the passivation, and at a still higher concentration (2000 ppm), the passivation characteristics were remarkably perturbed.

**Figure 13:**
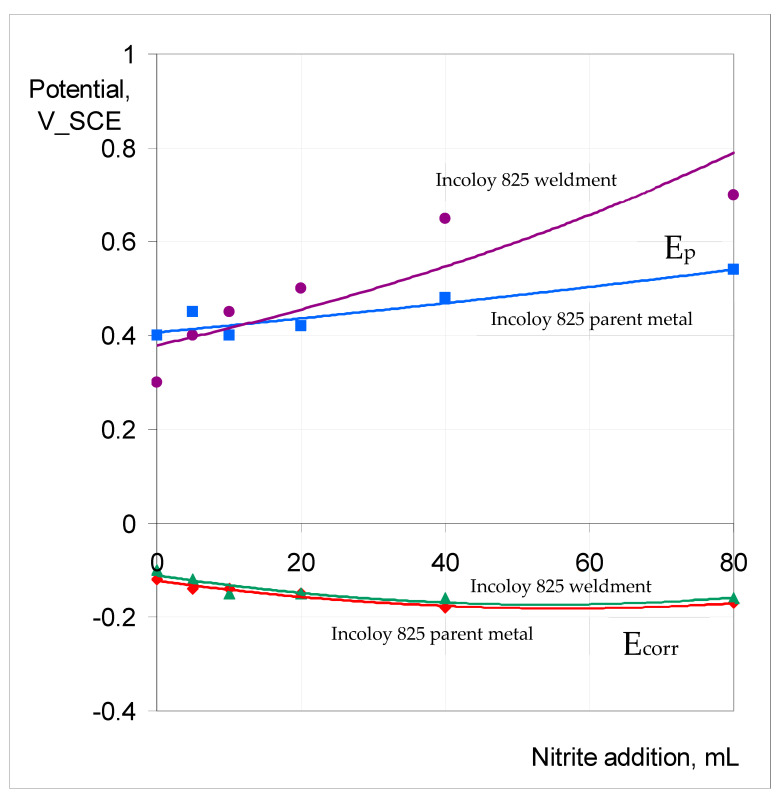
E_p_ and E_corr_ vs. nitrite concentration in 0.1 M NaCl for Incoloy 825 parent metal and weldment at 90 °C.

Figure 13 summarises the variation in E_p_ and E_corr_ for the parent metal and weldment of Incoloy 825 exposed to 0.1 M NaCl at 90 °C with increasing NaNO_2_ additions. Somewhat unlike SAF 2507, there was an observable trend of E_p_ ennoblement with the increasing nitrite content of 0.1 M NaCl, and the weldment underwent greater ennoblement. E_corr_ varied within 100 mV over the nitrite addition regime for both the parent metal and the weldment.

**Figure 14:**
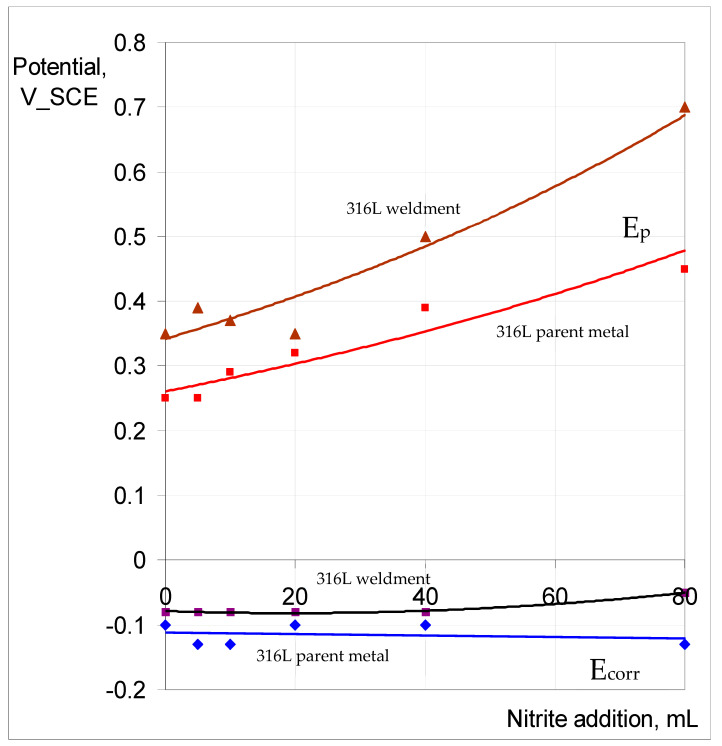
E_p_ and E_corr_ vs. nitrite concentration in 0.1 M NaCl for 316L parent metal and weldment at 60 °C.

The trend of increasing E_p_ ennoblement with nitrite content of 0.1 M NaCl, and the E_corr_ remaining relatively unchanged, as seen for the Incoloy 825 parent metal and weldment (Figure 13), was also observed for 316L (as shown in Figure 14). However, the E_p_ ennoblements were significantly more pronounced for both the weldment and base metal of 316L compared to the corresponding samples of Incoloy 825 (Figure 13 and Figure 14).

The passivation and pitting resistance characteristics of SAF 2507 first improved when increasing the nitrite content of the chloride solution and were compromised with a further increase in the nitrite content, and the trend is consistent with the reported literature [28]. The passivation and pitting resistance characteristics of 316L continued to improve with an increasing nitrite content of the chloride solution (Figure 14). Given the considerable difference in the pitting resistance of SAF and that of 316L, it seems it may require much higher nitrite content for the perturbance of the passivation to kick in.

## 4. Conclusions

This investigation was carried out as a result of an industrially observed pitting of the weldments of the duplex stainless steel SAF2507 in chloride solutions with a nitride addition, which was unexpected, given the reported high pitting resistance (and PREN) of this steel. The pitting and corrosion resistance of SAF2507 and its weldments were investigated in highly corrosive FeCl_3_, as well as in less corrosive NaCl solutions, with and without different levels of nitrite. The microstructures of the weldments were also characterised. To develop a comparative appreciation, the investigations also included another highly corrosion-resistant alloy, Incoloy 825, and a common austenitic stainless steel (316L). This work has established the influence of a NO_2_^−^ addition to the chloride solution in the susceptibility of the weldment of the alloys to chloride pitting.

The corrosion rate of the parent metal (3.5 × 10^3^ μg/m^2^ s) and weldment (6 × 10^2^ μg/m^3^ s) of SAF 2507 were respectively similar to those of 316L stainless steel in FeCl_3_ solutions with and without nitrite additions; however, SAF 2507 showed a greater resistance to pitting. The corrosion resistance of both the parent metal (5 × 10^2^ μg/m^2^ s) and weldment (1.5 × 10^3^ μg/m^2^ s) of Incoloy 825 was much lower (than that of SAF 2507). For each alloy, the corrosion rate of the parent metal was lower in each environment, which was attributed to the greater pitting susceptibility of the HAZ in the weldment of each alloy.

The role of nitrite additions to 0.1 M NaCl in critical pitting temperature (CPT) was characterised, and 316L showed a profound influence via nitrite additions in inhibiting pitting (i.e., an increase in pitting potential (E_p_) by 200 mV when nitrite was added to the electrolyte), whereas the nitrite addition caused very little effect on the corrosion potential, E_corr_ (i.e., around −100 mV vs. SCE). Also, ‘meta-stable’ pitting transients that were clearly visible in 0.1 M NaCl without nitrite were absent when nitrite was added to the test solution. Both the parent metal and weldment of SAF2507 had a similar E_p_ (~700 mV) in 0.1 M NaCl without nitrite, which was the highest E_p_ among the three alloys tested. The additions of 20 mL and 40 mL of 0.1 M NaNO_2_ had an inhibitive effect on pitting, whereas a further increase in the nitrite content (to 80 mL) compromised pitting resistance. On the other hand, Incoloy 825 showed a trend of E_p_ ennoblement with an increasing nitrite content of 0.1 M NaCl, and the weldment underwent greater ennoblement, whereas E_corr_ varied within 100 mV over the nitrite addition regime for both the parent metal and weldment. Moreover, 316L showed a trend similar to that of Incoloy 825; however, the E_p_ ennoblements were significantly more pronounced for both the weldment and base metal of 316L.

Given that pits are the most common initiators of stress corrosion cracking (SCC), it would be prudent to investigate the role of nitrite additions in the susceptibility of the weldments of the three alloys to SCC, utilising the data on the role of nitride additions to a chloride solution in the pitting susceptibility of the weldments of the alloys.

## Figures and Tables

**Table 1:** Chemical compositions (wt.%) of SAF 2507, Incoloy 825 and 316L SS.

Test Alloys	C	Cr	Ni	Mn	Mo	N	Cu	P	Si	Al	S	Fe
SAF2507	0.02	24.6	6.52	0.83	3.8	0.27	0.13	0.02	0.38	<0.001	<0.01	Balance
Typical composition of SAF2505	0.03	24.00–26.00	6.00–8.00	1.20	3.00–5.00	0.24–0.32	-	0.035	0.8	-	0.02	Balance
Incoloy 825	0.01	24.1	39.3	0.77	3.0	<0.001	1.6	<0.01	0.24	0.04	<0.01	Balance
Typical composition of Incoloy 825	0.05	19.50–23.50	38.00–46.00	1.00	2.50–3.50	-	1.50–3.00	-	0.5	0.2	0.03	Balance
316 L	0.02	16.6	10.0	1.69	2.1	0.04	0.39	0.03	0.39	<0.001	0.01	Balance
Typical composition of 316L	0.03	16.00–18.00	10.00–14.00	2.00	2.00–3.00	0.10	-	0.045	0.75	-	0.03	Balance

**Table 2:** Chemical compositions (wt.%) of welding filler wires: 2507 filler wire (AWS A5.9 ER25.10.4.L), Inconel 625 filler wire for Incoloy 825 (AWS A5.14 ERNiCrMo-3) and 316L filler wire (AWS A5.9 ER316LSi).

Material	Ni	Cr	Mo	C	Cu	N	Mn	Ti	S	Si	P	Fe
2507	9.5	25	4	0.02	-	0.25	0.4	-	-	0.35	-	Bal
Inconel 625	58 (Ni+Co)	20–23	8–10	0.1	0.5	-	0.5	0.4	0.015	0.5 (+0.4 Al)	-	Bal
316L	11–14	18–20	2–3	0.03	-	-	1–2.5	-	0.03	-	0.03	Bal

**Table 3:** Details of the etching process used in the electrochemical etching of the three alloys.

Materials	Etchant	Voltage (V)	Current (A)	Time (s)	Observations
SAF 2507	10% (wt.) oxalic acid	20	5	100	Satisfactory microstructure
Incoloy 825	10% (wt.) oxalic acid	15	5	20	Over-etched, grain boundaries depleted
SS 316L	10% (wt.) oxalic acid	20	5	100	Satisfactory microstructure
SAF 2507	10 g chromic acid in 100 mL water	10	5	30	Satisfactory microstructure
Incoloy 825	10 g chromic acid in 100 mL water	10	5	30	Satisfactory microstructure
SS 316L	10 g chromic acid in 100 mL water	6	5	50	Satisfactory microstructure

**Table 4:** Nitrite concentration in 6% FeCl_3_ solution for weight loss measurements.

Chloride:Nitrite Ratio	Nitrite Concentration (ppm)	Mass of NaNO_2_ Required (g) per 200 mL 6% FeCl_3_ *
10:1	3963	1.18
6.667:1	5903	1.77
5:1	7872	2.36

* Chloride concentration in 6% wt. FeCl_3_ solution is 39,360 ppm.

**Table 5:** Calculated PREN numbers for SAF 2507, Incoloy 825 and 316L.

Elements	Cr	Mo	N	PREN
SAF 2507	24.78	3.78	0.27	41.6
Incoloy 825	22.45	3.22	<0.001	33.1
316L	16.52	3.22	0.04	27.8

## Data Availability

The study’s data are presented within the article.

## Abbreviations

SSs	Stainless steels
DSSs	Duplex stainless steels
ASSs	Austenitic stainless steels
E_p_	Pitting potential
SCC	Stress corrosion cracking
PREN	Pitting resistance equivalent number
IGC	Intergranular corrosion
PDP	Potentiodynamic polarisation
HAZ	Heat-affected zone
CPT	Critical pitting temperature

## References

[1] Kwon H.-S., Kim H.-S. (1993). Investigation of stress corrosion susceptibility of duplex (α + γ) stainless steel in a hot chloride solution. Mater. Sci. Eng. A.

[2] Shimodaira S., Takano M., Taleizawa Y., Kamida H. (1977). SCC and Hydrogen Embrittlement of Iron-Base Alloys.

[3] Sedriks A.J. (1996). Corrosion of Stainless Steels.

[4] Egan F., Clapp G. In Proceedings of the Corrosion & Prevention, Hobart, Australia, 23–25 November 1998; paper#38-066, pp. 1–6.

[5] Notten M.J.G. (1991). Experiences with ferritic-austenitic stainless steel in chemical process industry—Duplex are not a panacea. Stainl. Steel Eur..

[6] Jones D.A. (1992). Principles & Prevention of Corrosion.

[7] Raman R.K.S. (2006). Role of caustic concentration and electrochemical potentials in caustic cracking of steels. Mater. Sci. Eng. A.

[8] Singbeil D., Tromans D. (1981). Effect of Sulfide Ions on Caustic Cracking of Mild Steel. J. Electrochem. Soc..

[9] Singbeil D., Tromans D. (1982). Caustic Stress Corrosion Cracking of Mild Steel. Metall. Transact. A.

[10] Francis R., Gooch T.G. (1994). Duplex Stainless Steels ’94.

[11] Charles J., Bernhardsson S. (1991). Duplex Stainless Steel ’91.

[12] Truman J.E., Kirby H.W. (1965). The Possibility of Service Failure of Stainless Steels by Stress Corrosion Cracking. Metallurgia.

[13] Ward I.A., Keys L.H. (1984). Stainless Steels ’84.

[14] Nilsson J.O., Wilson A. (1993). Influence of isothermal phase transformations on toughness and pitting corrosion of super duplex stainless steel SAF 2507. Mater. Sci. Technol..

[15] Cottis R.A., Newman R.C. (1995). SCC of Duplex Stainless Steels, Health and Safety Executive—Offshore Technology Report.

[16] Newman R.C., Shahrabi T. (1987). The effect of alloyed nitrogen or dissolved nitrate ions on the anodic behaviour of austenitic stainless steel in hydrochloric acid. Corros. Sci..

[17] Newman R.C., Ajjawi M.A. (1986). A micro-electrode study of the nitrate effect on pitting of stainless steels. Corros. Sci..

[18] Zuo Y., Wang H., Zhao J., Xiong J. (2002). The effects of some anions on metastable pitting of 316L stainless steel. Corros. Sci..

[19] Uhlig H.H., Cook J.E.W. (1969). Mechanism of Inhibiting Stress Corrosion Cracking of 18-8 Stainless Steel in MgCl_2_ by Acetates and Nitrates. J. Electrochem. Soc..

[20] Leckie H.P., Uhlig H.H. (1975). Environmental Factors Affecting the Critical Potential for Pitting in 18–8 Stainless Steel. J. Electrochem. Soc..

[21] Jones R.L. (1975). Nitrate Effects Promoting the Stress Corrosion Cracking of AISI Type 304 Stainless Steel in MgCl_2_ Above 200 C. Corrosion.

[22] Illevbare G.O., King K.J., Gordon S.R., Elayat H.A., Gdowski G.E., Summers T.S.E. Effect of nitrate on the repassivation potential of alloy 22 in chloride containing environments. Proceedings of the 206th Meeting of The Electrochemical Society.

[23] Refaey S.A.M., El-Rehim S.S.A., Taha F., Saleh M.B., Ahmed R.A. (2000). Inhibition of chloride localized corrosion of mild steel by PO_4_^3−^, CrO_4_^2−^, MoO_4_^2−^, and NO_2_^−^ anions. Appl. Surf. Sci..

[24] Bardwell J.A., Sproule G.I., Mitchell D.F., Macdougall B., Graham M.J. (1991). Nature of the passive film on Fe–Cr alloys as studied by ^18^O secondary ion mass spectrometry: Reduction of the prior film and stability to ex situ surface analysis. J. Chem. Soc. Faraday Trans..

[25] Raman R.K.S., Siew W.H. (2014). Stress corrosion cracking of an austenitic stainless steel in nitrite-containing chloride solutions. Materials.

[26] Verma J., Taiwade R.V. (2017). Effect of welding processes and conditions on the microstructure, mechanical properties and corrosion resistance of duplex stainless steel weldments—A review. J. Manuf. Process..

[27] Baeslack W.A., Savage W.F., Duquett D.J. (1979). The Effect of Strain Rate on Stress Corrosion Cracking in Duplex Type 304 Stainless Steel Weld Metal. Metall. Trans..

[28] Raman R.K.S., Siew W.H. (2010). Role of nitrite addition in chloride stress corrosion cracking of a super duplex stainless steel. Corros. Sci..

